# Estrogen Enhances the Expression of the Polyunsaturated Fatty Acid Elongase *Elovl2* via ERα in Breast Cancer Cells

**DOI:** 10.1371/journal.pone.0164241

**Published:** 2016-10-27

**Authors:** Amanda González-Bengtsson, Abolfazl Asadi, Hui Gao, Karin Dahlman-Wright, Anders Jacobsson

**Affiliations:** 1 Department of Molecular Biosciences, The Wenner-Gren Institute, Stockholm University, Stockholm, Sweden; 2 Department of Biosciences and Nutrition, Karolinska Institutet, Huddinge, Sweden; Turun Yliopisto, FINLAND

## Abstract

Endocrine therapy is the first-line targeted adjuvant therapy for hormone-sensitive breast cancer. In view of the potential anticancer property of the omega-3 polyunsaturated fatty acid docosahexaenoic acid (DHA) together with chemotherapy in estrogen receptor alpha (ERα) positive mammary tumors, we have explored the regulation by estradiol of the fatty acid desaturation and elongation enzymes involved in DHA synthesis in the human breast cancer cell line MCF7, which expresses ERα but not ERβ. We demonstrate a robust up-regulation in the expression of the fatty acid elongases *Elovl2* and *Elovl5* upon estradiol stimulation in MCF7 cells, which was sustained for more than 24 hours. Exposure with the ER inhibitor tamoxifen abolished specifically the *Elovl2* but not the *Elovl5* expression. Similarly, knock-down of ERα eliminated almost fully the *Elovl2* but not the *Elovl5* expression. Furthermore, ERα binds to one specific ERE within the *Elovl2* enhancer in a ligand dependent manner. The involvement of ERα in the control of especially *Elovl2*, which plays a crucial role in DHA synthesis, may have potential implications in the treatment of breast cancer.

## Introduction

Docosahexaenoic acid (DHA, 22:6) is an omega-3 polyunsaturated fatty acid (PUFA), which is abundant in fatty fish and other marine sources and has been shown to have a variety of health benefits on breast cancer both in rodents and humans [1] as well as in cell lines [2]. However, while the effects of dietary DHA have been extensively studied, less attention has been paid to the physiological role of endogenous DHA synthesis

**Fig 1:**
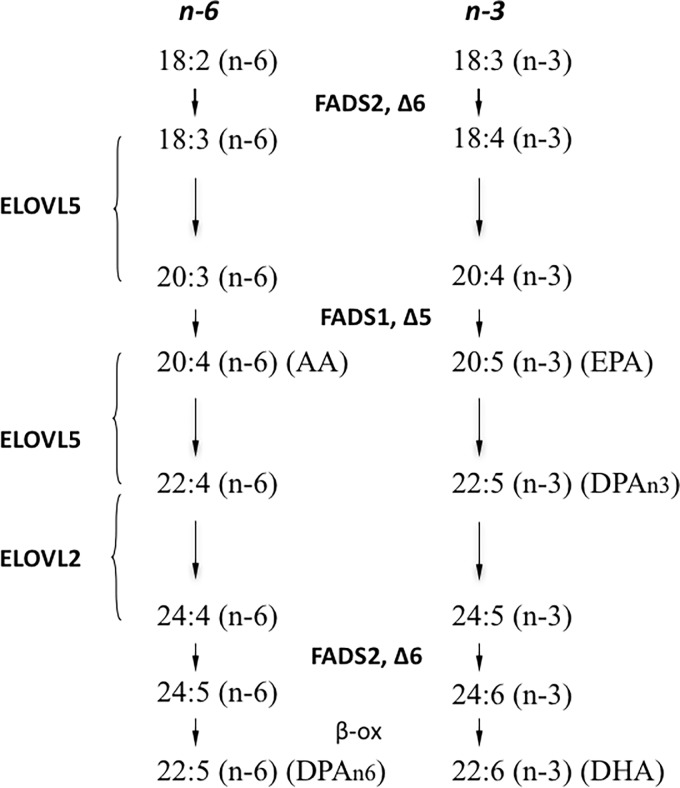
Schematic pathway of polyunsaturated fatty acid (PUFA) synthesis. The map shows the elongation and desaturation steps of omega 3 (n-3) and omega 6 (n-6) fatty acids connected to the major actions of ELOVL5, ELOVL2, FADS1 and FADS2.

Synthesis of omega 3 and omega 6 PUFAs is accomplished by sequential elongation and desaturation steps of the essential fatty acids linoleic acid (C18:2 n-6) and α-linolenic acid (C18:3 n-3) and their derivatives [3] (Fig 1). The involved enzymes are located in the endoplasmatic reticulum and include the fatty acid desaturases 1 (FADS1) and 2 (FADS2) and the fatty acid elongases elongation of very long-chain fatty acids 2 (ELOVL2) and 5 (ELOVL5) where ELOVL2 is considered to be essential for the formation of C24 PUFAs in a tissue specific manner prior to further desaturation and β-oxidation of 24:6n-3 into DHA [4–6].

The connection between steroidal hormones such as estrogen and PUFA synthesis has previously been studied showing that hepatic *Fads2* expression was up regulated in response to increased progesterone and 17-β-estradiol (E2) concentrations in female rats, followed by increased levels of long chain PUFAs [7]. Estrogen, after binding to estrogen receptors (ERs), regulates gene expression through interaction with specific estrogen response elements (ERE) within DNA [8,9]. ERs are part of the nuclear receptor superfamily of transcription factors and have important implications in hormone-related disorders, development and physiology [10]. ERs exist as two different subtypes; ERα and ERβ [11], which have the ability to form heterodimers [12] as well as homodimers [13]. The DNA binding domains (DBDs) of the receptors are 97% homologous [14,15] and particularly the P-box, which is essential for DNA specificity, is 100% identical [16]. In line with this, ERα and ERβ has been shown to bind to a diverse range of EREs with similar selectivity and affinity [12,13]. There is a wide diversity of ER ligands with varying affinity. The endogenous ligand E2 binds with similar affinity to both ERα and ERβ [17].

ERα enhances proliferation of endocrine responsive breast cancers, while ERβ in several studies exerts an inhibitory action on cancer cell growth [18,19]. As approximately 80% of all breast cancers are ERα positive, endocrine therapy is considered complementary to surgery in the majority of patients [20].

To determine how estrogen via ERα effects enzymes involved in PUFA synthesis, we have examined the expression of desaturases and elongases in ERα positive MCF7 cells and ERα negative HepG2 cells upon E2 treatment. We show that E2 primarily stimulates the expression of *Elovl2* and *Elovl5* in MCF7 cells and that ERα directly binds to one specific ERE within the *Elovl2* promoter upon estrogen stimulation in MCF7 cells.

## Materials and Methods

### Cell culture

The human breast cancer cell line MCF7 was cultured in Minimum Essential Medium (ATCC) supplemented with 10% FBS and 0,5% Penicillin-Streptomycin. The human liver hepatocellular carcinoma cell line HepG2 was cultured in Dulbecco’s modified medium with 10% FBS and 1% Penicillin-Streptomycin. Both cell lines were cultured in 6 well plates, apart from the ChIP experiments (see below), and kept at 37°C in 5% CO_2_. Before treatment, the cells were cultured in RPMI 1640 phenol free medium containing 2% charcoal treated FBS and 0,5% Penicillin-Streptomycin for 72 hours. All material/chemicals were purchased from Sigma Aldrich except ICI 182,780 (TOCRIS bioscience).

### ERα and ERβ overexpressing cells

MCF7 and HepG2 cells were transiently transfected for 24 hours with different amounts of pcDNA3 expression vectors containing ERα and ERβ using Lipofectamine 2000 (Invitrogen). Cells were then exposed to 10 nM E2 or vehicle (ethanol) for 6 hours and then harvested for RNA preparation.

### Transient knock-down of ERα

MCF-7 cells seeded in 6-well plates were maintained in phenol red-free DMEM supplemented with 5% charcoal treated FBS for 48 hr. Cells were transiently reverse transfected with 50 nM of either control siRNA or ERα siRNA (siRNA-A: sc-37007, h: sc-29305 Santa Cruz Biotechnology, Inc., Santa Cruz, CA) using Lipofectamine RNAiMAX Transfection Reagent (ThermoFisher Scientific catalg number: 13778075.) according to the manufacturer's instructions. After 48 hr, the cells were serum starved for 12 hr and either treated with 10 nM E2 or vehicle (ethanol) for 4 hr. The protein expression of ERα was determined by Western blot and the mRNA expression of *Elovl2* and *Elovl5* was measured by qPCR.

### Real-time PCR analysis

Real-Time PCR was performed with SYBR Green JumpStart Taq ReadyMix for QPCR from Sigma Aldrich. To investigate the expression of the indicated genes, total RNA was isolated with TRIReagent (Sigma Aldrich) following manufacture’s procedure. For real time PCR, 500 ng of total RNA was reverse transcribed using random hexamer primers, dNTPs, multiscript and RNase inhibitor (Applied Biosystems, Foster City, CA, USA). cDNA samples were diluted 1:10 and aliquots of 2μl were mixed with SYBR Green JumpStart Taq ReadyMix (Sigma Aldrich), pre-validated primers, DEPC treated water and were analysed in triplicate for each sample. For primer sequences used to detect *ERα* and *ERβ*, *Elovl2*, *Elovl5*, *Fads1* and *Fads2*, see S1 Table. PCR products were detected using a BioRad detection system. Data were normalized to the housekeeping gene 36B4.

### Western blotting

For immunoblotting of ERα, cells were lysed in Ripa buffer and proteins from each sample (10 μg/lane) were separated by 12% SDS-PAGE and blotted to a polyvinylidene difluoride transfer membrane (Amersham Hybond-P; GE Healthcare) in a semidry system. The membrane was incubated for 1 hour in 5% fat-free milk, then overnight with the diluted 1:1000 primary ERα antibody (Hc-20: sc-543; Santa Cruz Biotechnology). Bound antibodies were detected with a secondary peroxidase-conjugated anti-rabbit (anti-rabbit; Cell Signaling) diluted 1:2000 in 5% fat-free milk, 10× TBS, and Tween-20. The membranes were washed with TBS and Tween-20 2× for 5 min and 15 min, respectively, after each incubation time. Proteins were visualized using an ECL Plus kit (Amersham Bioscience) and detected in an LAS-1000 CCD camera (Fuji). Membranes were mild stripped by using a volume of buffer (15 g glycine, 1 g SDS, 10 ml Tween 20 dissolved in 1 L distilled water adjust PH to 2.2) that covered the membrane and incubated at room temperature for 10 min. The incubation was repeated and the membranes were, washed twice in TBS-T and PBS for 10 min each and re-probed with anti- β-actin monoclonal antibody (β-actin 13E5, Cell Signaling Technology) diluted 1:1000 in TBS-T, to serve as a loading control.

### Chromatin immunoprecipitation

MCF-7 cells were seeded in 15 cm culture dishes and reached 80–90% confluence after 3 days. Then they were treated with 10 nM E2 or vehicle (ethanol) for 45 min and ChIP was performed according to the previously published procedure [21]. Briefly, the cells were first fixed and DNA-protein cross linked with 1% formaldehyde. Cross-linking was quenched by adding 125 mM glycine and cells were then harvested and resuspended in lysis buffer [50 mM Tris-HCl (pH 8.0); 150 mM NaCl; 1 mM EDTA; 1% Triton X-100; 0.1% Na-deoxycholate] containing protease inhibitors (Roche, Mannheim, Germany). The soluble chromatin was obtained by sonication and were incubated with 30 μl ERα antibody (HC20; Santa Cruz) coupled magnetic beads (Invitrogen, USA) or IgG under gentle agitation for overnight at 4°C. The beads pellets were successively washed for 3 min in 1 ml buffer 1 [20 mM Tris-HCl (pH 8.0); 150 mM NaCl; 2 mM EDTA; 1% Triton X-100; 0.1% sodium dodecyl sulfate (SDS)], 1 ml buffer 2 [20 mM Tris-HCl (pH 8.0); 500 mM NaCl; 2 mM EDTA; 1% Triton X-100; 0.1% SDS], 1 ml LiCl buffer [20 mM Tris-HCl(pH 8.0); 250 mM LiCl; 1 mM EDTA; 1% Nonidet P-40; 1% Na-deoxycholate] and 2× 1 ml TE [10 mm Tris-HCl (pH 8.0); 1 mM EDTA]. Protein:DNA complexes were eluted in 120 μl elution buffer [1% SDS, 0.1M NaHCO] for 30 min, and the cross-links were reversed by overnight incubation at 65°C. DNA was purified using a PCR purification kit (QIAGEN, Valencia, CA) and eluted in 50 μl. Using the first five thousands bp upstream of the human *Elovl2* transcription start site, the predicted ERE binding sites of Elovl2 was obtained from the Transcriptional Regulatory Element Database via IUPAC/Regular Expression Analysis Results. The recruitments of ERα to the predicted ERE were detected by PCR using the ChIPped-DNA. Sequences of the PCR primers used are given in S3 Fig.

### Statistical analysis

Statistical analysis was performed using GraphPad PRISM (San Diego, CA) and statistical differences were calculated with Student’s unpaired t-test. Bars indicate mean ± SE *P<0.05, **P<0.01, ***P<0.001

## Results

### E2 stimulates *Elovl2* and *Elovl5* expression in MCF7 cells

**Fig 2:**
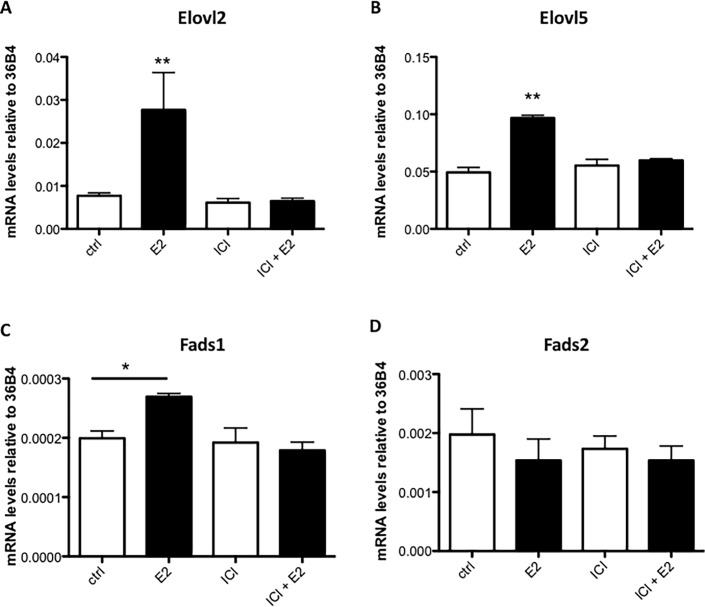
The effect of E2 treatment on the expression of PUFA synthesis enzymes in MCF7 cells. MCF7 cells were treated with 10 nM E2 and/or 10 μM ICI182,780 compared or vehicle (ethanol) for 6 hours. *Elovl2*, *Elovl5*, *Fads2* and *Fads1* mRNA levels (A, B, C and D) were determined by quantitative RT-PCR and normalized to the reference gene 36B4. Results shown are means ± SE of two individual experiments in triplicate. Statistical significances are indicated as *P<0.05 and **P<0.01.

**Fig 3:**
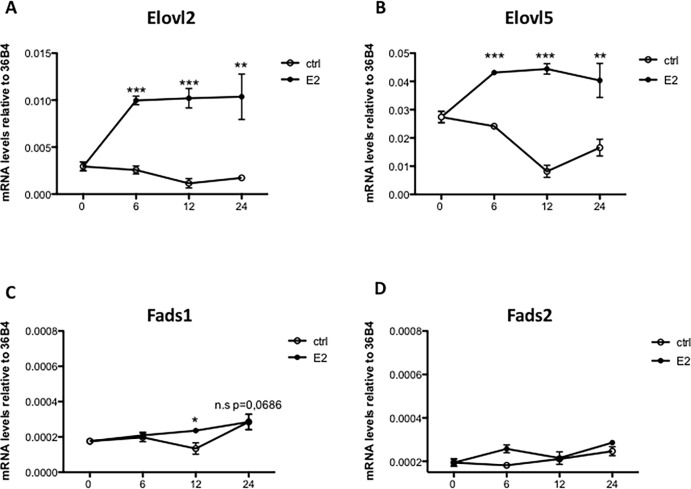
E2 time-response of PUFA synthesizing enzyme expression in MCF7 cells. MCF7 cells were treated with 10 nM E2 or vehicle (c) for 0, 6, 12 or 24 hours and *Elovl2*, *Elovl5*, *Fads1* and *Fads2* mRNA levels (A, B, C and D) were determined by quantitative RT-PCR and normalized to the reference gene 36B4. Results shown are means ± SE of two individual experiments in triplicate. Statistical significances are indicated as *P<0.05 and **P<0.01.

To assess if estrogen has the potential to regulate PUFA and especially DHA synthesis via regulating the expression of *Elovl2*, *Elovl5*, *Fads1* and *Fads2*, MCF7 cells were treated for six hours with the ER ligand E2. As shown in Fig 2, *Elovl2* and *Elovl5* expression were up-regulated approximately 5 fold and 2 fold, respectively, by E2 (Fig 2A and 2B). The expression of *Fads1* was slightly induced by E2, although the expression level was very low (Ct values 32–33) while the expression of *Fads2* was unaffected (Fig 2C and 2D). Administration of the ERα antagonist ICI 182780 one hour before E2 exposure attenuated the estrogen response whilst the antagonist alone had no effect (Fig 2). Furthermore, the increase in *Elovl2* and *Elovl5* mRNA levels by E2 was sustained for more than 24 hours (Fig 3A and 3B), which implies that estrogen via ERα has the potential to induce PUFA synthesis primarily via up-regulation of the elongases *Elovl2* and *Elovl5*.

### Tamoxifen supresses *Elovl2* but not *Elovl5* expression

**Fig 4:**
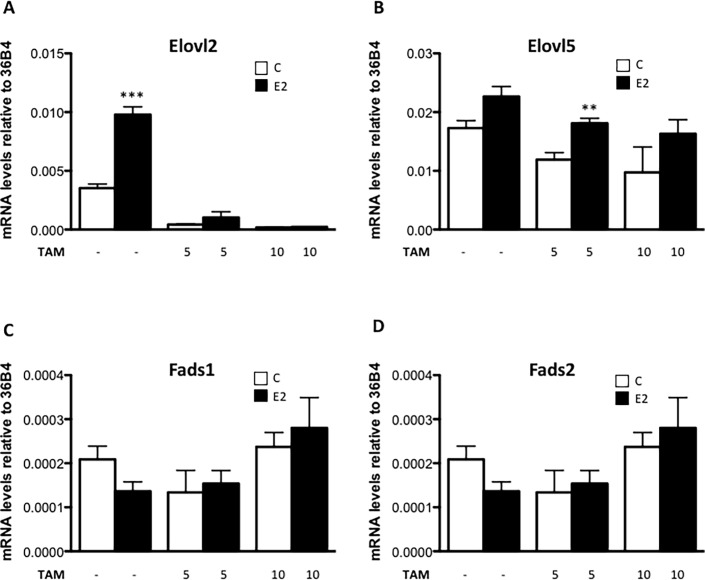
The effect of tamoxifen on PUFA synthesizing enzyme expression in MCF7 cells. MCF7 cells were treated with 5 μM or 10μM tamoxifen with and without 10 nM E2 or vehicle (c) for 24 hours and *Elovl2*, *Elovl5*, *Fads1* and *Fads2* mRNA levels (A, B, C and D) were determined by quantitative RT-PCR and normalized to the reference gene 36B4. Results shown are means ± SE of two individual experiments in triplicate. Statistical significances are indicated as *P<0.05, **P<0.01 and ***P<0.001.

Tamoxifen is an ER antagonist used for reducing the harmful effects in patients with hormone receptor positive breast cancer. To investigate if tamoxifen modulates E2-induced expression of *Elovl2* and *Elovl5*, it was applied to MCF7 cells at different concentrations. Interestingly, 5 μM tamoxifen profoundly reduced basal *Elovl2* expression and completely abolished the E2 stimulation at a consentration of 10 μM (Fig 4A). The basal expression level of *Elovl5* was somewhat reduced although tamoxifen did not block the E2 effect (Fig 4B). On the contrary, *Fads1* and *Fads2* expression remained unchanged upon tamoxifen administration, once again demonstrating their non-responsiveness to estrogen receptor modulation (Fig 4C and 4D).

### Endogenous levels of ERα are sufficient for E2-dependent induction of *Elovl2* and *Elovl5* expression in MCF7 cells

**Fig 5:**
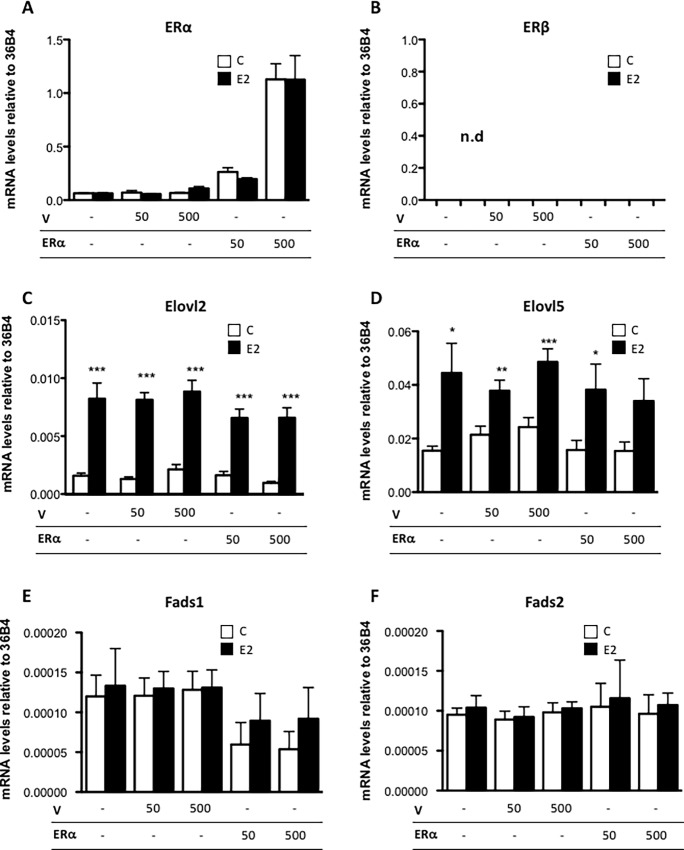
ERα overexpression does not modify the expression of PUFA elongases and desaturases in MCF7 cells. MCF7 cells were transfected with different concentrations (50ng or 500ng) of *ERα* or empty plasmid (V) as indicated for 24 hours followed by incubation with 10 nM E2 or vehicle (c) for 6 hours. (A) *ERα*, (B) *ERβ*, (C) *Elovl2*, (D) *Elovl5*, (E) *Fads1* and (F) *Fads2* mRNA expression were determined by quantitative RT-PCR normalized to the reference gene 36B4. Results shown are means ± SE of three individual experiments in triplicate. Statistical significances are indicated as *P<0.05, **P<0.01 and ***P<0.001.

To determine if the ERα level is a limiting factor for E2 regulation of *Elovl2* and *Elovl5* expression in MCF7 cells, ERα was overexpressed by transfecting ERα at different concentrations into the cells. Overexpression led to significantly higher levels of ERα transcript compared to cells treated with empty vector, with the highest expression level observed upon transfection of 500 ng expression vector (Fig 5A). We also confirmed that there was no detectable ERβ expression in MCF7 cells as previously reported [22] (Fig 5B). Upon exposure to E2, both *Elovl2* and *Elovl5* expression were increased independent of the amount of transfected ERα (Fig 5C and 5D), suggesting that the endogenous levels of ERα are sufficient for maximal E2-dependent induction of *Elovl2* and *Elovl5* expression in MCF7 cells (Fig 2A and 2B). In contrast, E2 did not induce *Fads1* and *Fads2* expression at any of the investigated ERα expression levels. However, ERα overexpression appeared to reduce *Fads1* expression independent of E2 although this was not statistically significant (Fig 5E).

### ERα and E2 independent expression of *Elovl2*, *Elovl5*, *Fads1 and Fads2* in HepG2 cells

To study if the expression of the PUFA enzymes could be stimulated by estrogen signaling in HepG2 cells,which is a human liver cell line that neither expresses ERα nor ERβ but expresses all the PUFA-synthesizing enzymes, ERα was transiently transfected, followed by ligand administration (S1A Fig). As seen in S1C–S1F Fig, ERα overexpression alone or in combination with E2 did not effect the expression of *Elovl2*, *Elovl5*, *Fads1* or *Fads2*.

### ERβ overexpression does not modify the expression of enzymes connected with PUFA synthesis

**Fig 6:**
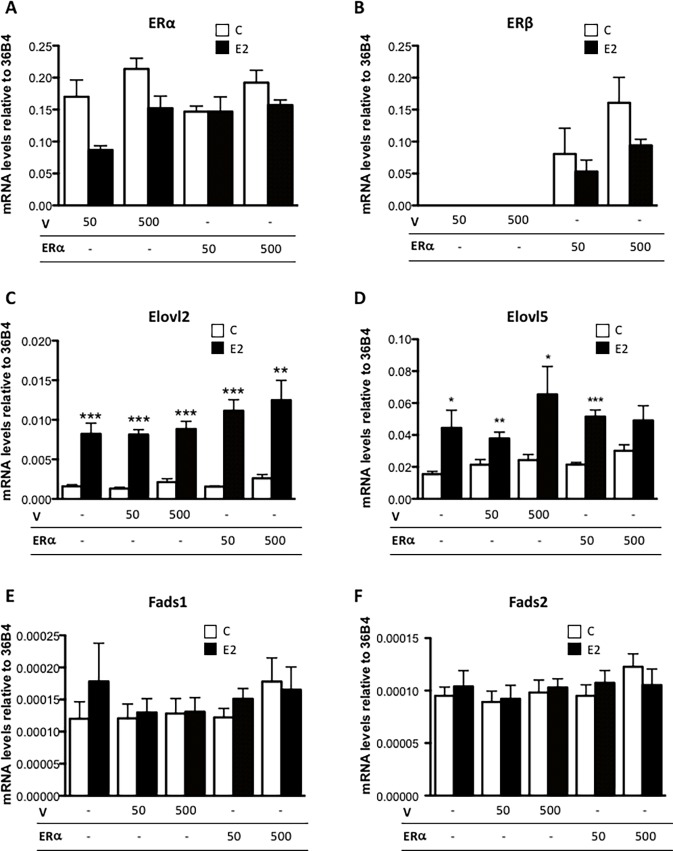
ERβ overexpression did not influence the expression of PUFA elongases and desaturases in MCF7 cells. A) MCF7 cells were transfected with different concentrations (50ng or 500ng) of *ERβ* or empty plasmid (V) as indicated for 24 hours followed by incubation with 10 nM E2 or vehicle (c) for 6 hours. (A) *ERα*, (B) *ERβ*, (C) *Elovl2*, (D) *Elovl5*, (E) *Fads1* and (F) *Fads2* mRNA expression were determined by quantitative RT-PCR normalized to the reference gene 36B4. Results shown are means ± SE of two individual experiments in triplicate. Statistical significances are indicated as *P<0.05, **P<0.01 and ***P<0.001, n.d = not detectable.

Since ERβ shares DNA binding specificity with ERα we examined the potential of ERβ to alter the expression of the PUFA synthesizing enzymes in MCF7 (Fig 6) and HepG2 (S2 Fig) cells, that lack endogenous ERβ expression. As seen in Fig 6B and S2B Fig, ERβ transfection caused significantly increased levels of ERβ mRNA levels compared to cells transfected with empty plasmid, with the highest expression level observed with 500 ng expression vector for both MCF7 and HepG2 cells. E2 significantly induced *Elovl2* and *Elovl5* expression via ERα in MCF7 cells but not in HepG2 cells as expected (Fig 6C and 6D and S2C and S2D Fig). However, the expression of the PUFA enzymes in both cell lines was independent of the amount of ERβ transcript (Fig 6C–6F and S2C–S2F Fig) implying ERα as the sole estrogen receptor in the control of PUFA synthesis in breast cancer cells.

### ERα knock-down supresses *Elovl2* but not *Elovl5* expression

**Fig 7:**
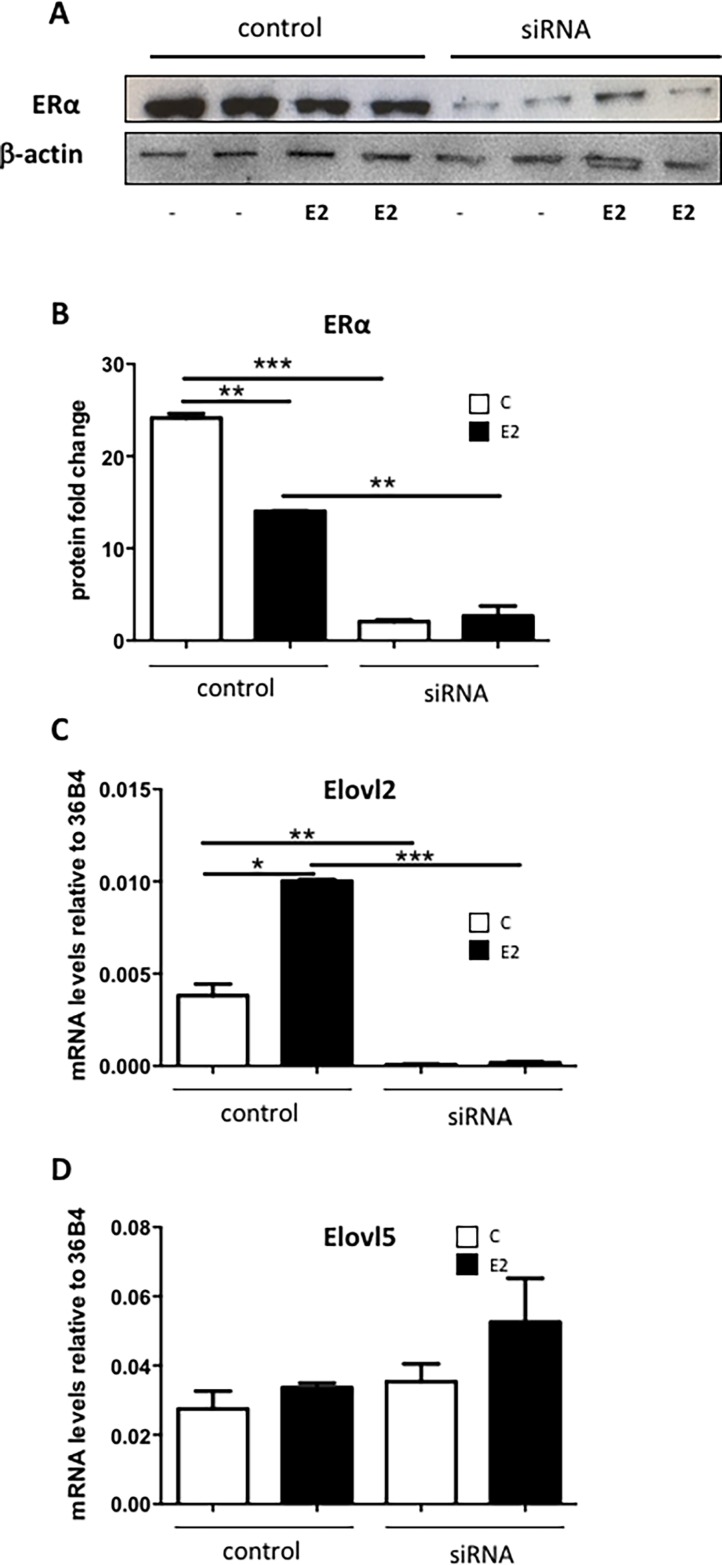
ERα knock-down reduces *Elovl2* expresion in MCF7 cells. MCF7 cells were transiently transfected with 50 nM ERα siRNA or siRNA control for 48 hours followed by incubation with 10 nM E2 (black bars) or vehicle (white bars) for 4 hours. (A) Western blot analysis using total protein extracts was performed to assess ERα and β-actin (control) protein levels. (B) Quantification of ERα protein levels, and (C) *Elovl2* and (D) *Elovl5* mRNA expression levels determined by quantitative RT-PCR normalized to the reference gene 36B4. Results shown are means ± SE of two individual experiments in duplicate. Statistical significances are indicated as *P<0.05, **P<0.01 and ***P<0.001.

To further validate the requirement of the presence of ERα for E2 induced *Elovl2* expression, a transient siRNA knock-down of ERα was performed in MCF7 cells. As seen in Fig 7A and 7B, ERα protein levels was significantly reduced by the siRNA treatment. In control cells, E2 exposure lead to a slight reduction of ERα, which is in accordance with previous reports [23]. The *Elovl2* expression was elevated in the control MCF7 cells treated with E2 as previously shown (Fig 7C). However, the *Elovl2* expression was almost undetectable in the ERα abolished MCF7 cells regardless of E2 stimulation (Fig 7C). In contrast, *Elovl5* expression levels were not affected by ERα abolishment (Fig 7D) supporting that ERα controls the PUFA enzyme machinery mainly via *Elovl2* in MCF7 cells.

## The Elovl2 promoter contains an ERE that associates with ERα upon ligand activation

**Fig 8:**
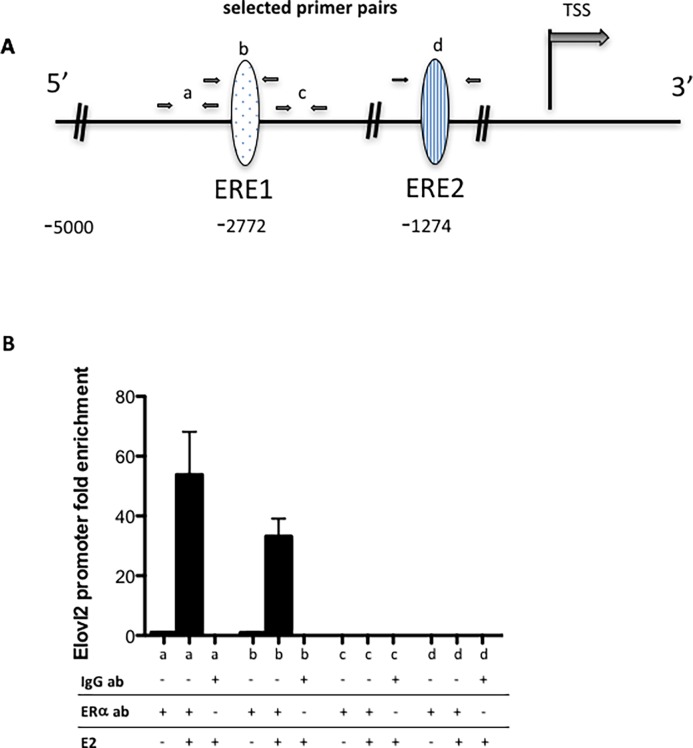
Binding of ERα to ChIP analysis of the *Elovl2* enhancer in MCF7 cells. (A) Schematic representation of two putative estrogen response elements, ERE1 and ERE2, within the *Elovl2* enhancer with the primer pairs used for ChIP assays illustrated as black arrows denoted a, b and c with the “a” primer pair positioned 5’ adjacent to the ERE1, the “b” primer pair covering the ERE1, the “c” primer pair 3’ of the ERE1 and the “d” primer pair covering the ERE2 site. (B) Fold difference for ERα and IgG (control) binding for E2 and vehicle treated MCF7 cells using primer pair a, primer pair b, primer pair c and primer pair d. Statistical significances are indicated as *P<0.05, **P<0.01 and ***P<0.001.

As ELOVL2 is a major factor in the control of cell specific DHA synthesis we investigated whether the stimulatory effect of estrogen on the expression of this gene was associated with direct ERα binding to the *Elovl2* gene. By using the “Transcriptional Regulatory Element Database” (Cold Spring Harbor Laboratory) two putative estrogen response elements (ERE) were identified, ERE1 and ERE2, located at– 2817 and– 1279, respectively, 5’ of the transcription start site within the *Elovl2* promoter (Fig 8A and S3 Fig). To determine whether ERα can bind to these EREs, a ChIP assay was performed. The different primer pairs designed to assess binding to each putative binding site are shown in Fig 8A and in S3 Fig. A clear enrichment could be detected at ERE1 in the presence of E2 (Fig 8B). Conversely, no enrichment of ERα binding could be detected at ERE2 (Fig 8B). This provides evidence that the ERE1 site in the *Elovl2* promoter is indeed interacting with ERα in breast cancer cells and that the association is ligand dependent.

## Discussion

Results of both *in vitro* and *in vivo* studies suggest that the omega-3 fatty acid (DHA) favourably modulates anticancer treatment responses by promoting cytotoxic effects and improving the effects of several anticancer drugs in different human cancer cells [24,25]. However, while the effects of dietary DHA have been extensively studied, research in the past shows that there is a significant genetic component linked to endogenous PUFA concentrations in humans indicating that understanding the regulation of enzymes involved in PUFA synthesis is of great physiological importance.

Here we have shown that the estrogen receptor agonist E2, which is the most prevalent estrogen in premenopausal women, functions as a potent stimulator of the expression of the PUFA elongases *Elovl2* and *Elovl5* but not the desaturases *Fads1* and *Fads2* in the human breast cancer cell line MCF7 and that the elevated expression was sustained for more than 24 hours.

We also demonstrate that tamoxifen, a typical anti-estrogen drug that is administered as first-line treatment for advanced breast cancer patients and for the prevention of breast cancer, selectively down regulates *Elovl2*, which implies that endogenous DHA production may be significantly affected in patients undergoing endocrine therapy. Interestingly, omega-3 fatty acid supplementation has previously been shown to attenuate the inherent apoptotic response of tamoxifen in MCF7 cells suggesting that a diet rich in omega-3 may diminish the beneficial effects of tamoxifen in breast cancer patients [26].

The ERα knock-down experiment cleary showed a selective down regulation of *Elovl2* expression. Interestingly, like tamoxifen, the ERα siRNA treatment almost abolished the *Elovl2* but not the *Elovl5* expression in control cells suggesting that prolonged anti-ERα-treatment has a negative effect on *Elovl2* expression which is E2 independent.

Our data also implies that the E2 effect on *Elovl2* expression involves direct binding of ERα to an ERE within the *Elovl2* enhancer. Furthermore, the findings suggest that the limiting factor for the induction of *Elovl2* expression in MCF7 cells is not the amount of ERα, but rather the availability of the ligand.

In contrary, the HepG2 cells, which normally are devoid of ERα, did not respond to E2 with regard to *Elovl2* expression when transfected with ERα suggesting that the transcription machinery controlling DHA synthesis is distinct for different cell types. This finding is consistent with a recent study showing that the mRNA levels of PUFA synthesis enzymes were not influenced in HepG2 cells in response to 17α–ethynylestradiol, a derivative of E2 [27].

On the basis of substrate competition studies, the n-6 and n-3 biosynthetic pathways involve the same desaturases and elongases [28]. However, our recent data on ELOVL2-ablated mice show that the major products of omega-6 and omega-3 synthesis in mammalian cells are arachidonic acid, 20:4n-6 (AA) and DHA (22:6n-3), respectively [6] and that ELOVL2 is the sole fatty acid elongase required for the elongation of 22-carbon PUFA C22:5n-3 into C24:5n-3 which is the essential precursors for DHA formation. While ELOVL5 and the desaturases FADS1 and FADS2 are expressed at significant levels in all tissues tested, ELOVL2 is highly expressed in liver, testis, uterus, placenta, mammary gland, and certain areas of the brain, all of which are tissues that are documented as being rich in DHA. In MCF7 cells, our data suggest that the expression levels of the elongases ELOVL2 and ELOVL5 are significantly higher than for the desaturases FADS1 and FADS2. This is in line with that MCF7 cells have been shown to have very low delta 6 desaturase activity [29]. The reason for this is unclear but is suggested to be a cause of a chromosome deletion in MCF7 cells [30]. However, our data show that both of the elongases, especially Elovl2, are controlled by E2 and ERα whereas the desaturases are not, which may have implications on PUFA levels in-vivo under certain conditions.

Despite this, our data showing that both of the elongases, especially ELOVL2, are controlled by E2 and ERα may have implications on PUFA levels in-vivo under certain conditions.

The activities of the fatty acid elongation enzymes are considered to be controlled at the transcriptional level [3,31]. Except ERα, it is still not known which other factors are involved in the control of *Elovl2* expression.

Upon estrogen binding, ERα undergoes a conformational change that facilitates the recruitment of coregulators to the promoter regions of target genes, either directly through interaction with cognate DNA sequences (ERE) or through protein/protein interaction with transcriptional binding sites [32,33]. Our data implies that the accessibility of ERα ligand is enough to enhance *Elovl2* expression and DHA formation in breast cancer cells.

Estrogen has prominent effects on breast cancer progression and malignancy [34]. Whilst ERα has been shown to bind numerous genes in breast cancer cells linked to developmental and proliferative functions [35], ERβ has been implicated as a potential tumor suppressor gene based on results that ERβ is lost in most of the breast cancers [36–38]. About two thirds of breast cancers in women require estrogen for growth, which is mainly mediated through ERα. The ERα-positive/ERβ-negative MCF7 breast cancer cells have been extensively studied and their gene expression profiles have been shown to strongly correlate with profiles in breast cancers *in vivo* [22]. However, the role of ERα activated *Elovl2* and Elovl5 expression and eventually increased DHA synthesis is unclear. A variety *in vitro* and animal analyses have revealed that breast cancer risk is associated with reduced omega-3/omega-6 ratio [39,40]. Furthermore, administration of the omega-3 PUFA DHA and EPA have been shown to inhibit cell proliferation and differentiation [41] and to activate apoptotic pathways in MCF7 cells [42,43]. Therefore, discerning the mechanism of action of ELOVL2, that has the potential to specifically modulate DHA availability, would be useful to unravel means to reduce cell viability in breast cancer.

We have previously shown that endogenous PUFA synthesis through ELOVL2 is the dominating factor to obtain systemic levels of DHA in mice [6]. As endocrine therapy is the standard targeted adjuvant therapy for hormone-sensitive breast cancer it would be relevant to investigate whether other cell types in the body, including liver that is one of the tissues with highest expression levels of *Elovl2* and DHA production, show diminished *Elovl2* expression upon anti ERα treatment. If so, this would highly support clinical dietary interventions with omega-3 fatty acids to potentially improve normal tissue functions in combination with deteriorating cancer progression in patients on endocrine therapy.

In summary, our data supports the notion that PUFA synthesis can be hormonally influenced in human breast cancer MCF7 cells by affecting the enzyme machinery responsible for PUFA elongation. Insights in the mechanisms whereby *Elovl2* mRNA levels can be regulated can have implications on DHA production and its protective effects in breast cancer patients.

## Supporting Information

S1 Fig ERα overexpression did not influence the expression of PUFA elongases and desaturases in HepG2 cells. HepG2 cells were transfected with different concentrations of *ERα* or empty plasmid (V) as indicated for 24 hours followed by incubation with 10 nM E2 or vehicle (c) for 6 hours. (A) *ERα*, (B) *ERβ*, (C) *Elovl2*, (D) *Elovl5*, (E) *Fads1* and (F) *Fads2* mRNA expression were determined by quantitative RT-PCR normalized to the reference gene 36B4. Results shown are means ± SE of two experiments in triplicate. No statistical significances are indicated as P>0.05, n.d = not detectable.
(PPTX)

S2 Fig ERβ overexpression did not influence the expression of PUFA elongases and desaturases in HepG2 cells. **A)** HepG2 cells were transfected with different concentrations of *ERβ* or empty plasmid (V) as indicated for 24 hours followed by incubation with 10 nM E2 or vehicle (c) for 6 hours. (A) *ERα*, (B) *ERβ*, (C) *Elovl2*, (D) *Elovl5*, (E) *Fads1* and (F) *Fads2* mRNA expression were determined by quantitative RT-PCR normalized to the reference gene 36B4. Results shown are means ± SE of two individual experiments in triplicate. No statistical significances are indicated as P>0.05, n.d = not detectable.
(PPTX)

S3 Fig Location and primer sequences used in ERα ChIP analysis of the Elovl2 enhancer. A) Four different primer pairs (a-d) and two putative estrogen response elements (ERE1 and ERE2), located at– 2817 to -2827 and -1279 to -1289, respectively, are indicated within the *Elovl2* promoter. B) a-d primer sequences An ERα ChIP assay was performed using four different primer pairs (a-d) as indicated (underlined) and B) sequences.
(PPTX)

S1 Table Primer sequences used for real time PCR analysis. (TIF)
