# Supplementary material for: Estrogen Enhances the Expression of the Polyunsaturated Fatty Acid Elongase Elovl2 via ERα in Breast Cancer Cells
Source: PLoS One. 2016 Oct 27;11(10):e0164241. doi: 10.1371/journal.pone.0164241 (PMC5082882; doi:10.1371/journal.pone.0164241)
Supplement: S2 Fig — A) HepG2 cells were transfected with different concentrations of ERβ or empty plasmid (V) as indicated for 24 hours followed by incubation with 10 nM E2 or vehicle (c) for 6 hours. (A) ERα, (B) ERβ, (C) Elovl2, (D) Elovl5, (E) Fads1 and (F) Fads2 mRNA expression were determined by quantitative RT-PCR normalized to the reference gene 36B4. Results shown are means ± SE of two individual experiments in triplicate. No statistical significances are indicated as P>0.05, n.d = not detectable. (PPTX) [file pone.0164241.s002.pptx]

## Slide 1
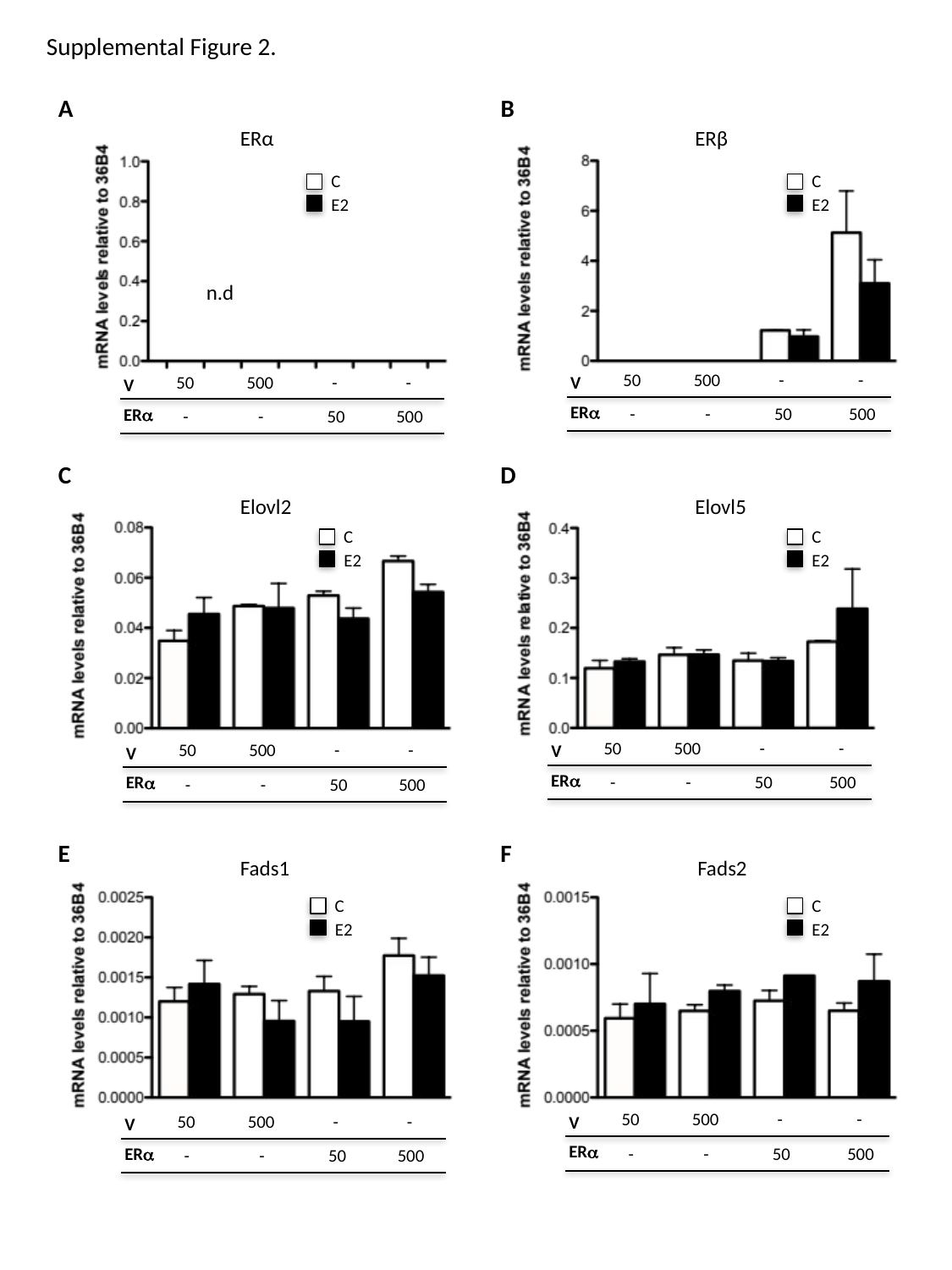

Supplemental Figure 2.
A
B
ERα
ERβ
C
E2
C
E2
n.d
50
-
500
-
-
50
-
500
V
ERa
50
-
500
-
-
50
-
500
V
ERa
C
D
Elovl2
Elovl5
C
E2
C
E2
50
-
500
-
-
50
-
500
V
ERa
50
-
500
-
-
50
-
500
V
ERa
E
F
Fads1
Fads2
C
E2
C
E2
50
-
500
-
-
50
-
500
V
ERa
50
-
500
-
-
50
-
500
V
ERa
