# Supplementary figures and images for: Estrogen Enhances the Expression of the Polyunsaturated Fatty Acid Elongase Elovl2 via ERα in Breast Cancer Cells
Source: PLoS One. 2016 Oct 27;11(10):e0164241. doi: 10.1371/journal.pone.0164241 (PMC5082882; doi:10.1371/journal.pone.0164241)

## Slide 1
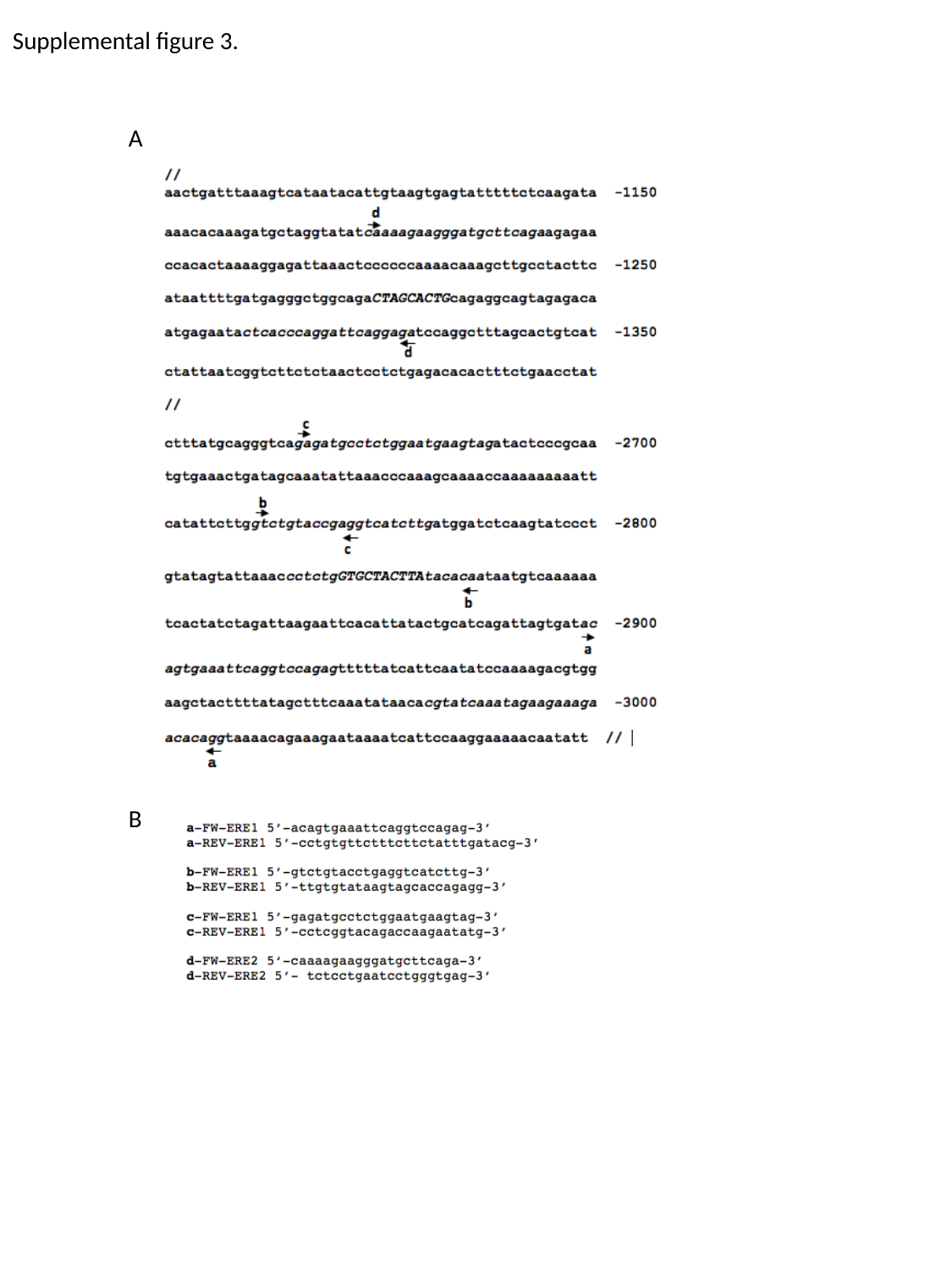

Supplemental figure 3.
A
B

Supplement: S3 Fig — A) Four different primer pairs (a-d) and two putative estrogen response elements (ERE1 and ERE2), located at– 2817 to -2827 and -1279 to -1289, respectively, are indicated within the Elovl2 promoter. B) a-d primer sequences An ERα ChIP assay was performed using four different primer pairs (a-d) as indicated (underlined) and B) sequences. (PPTX) [file pone.0164241.s003.pptx]

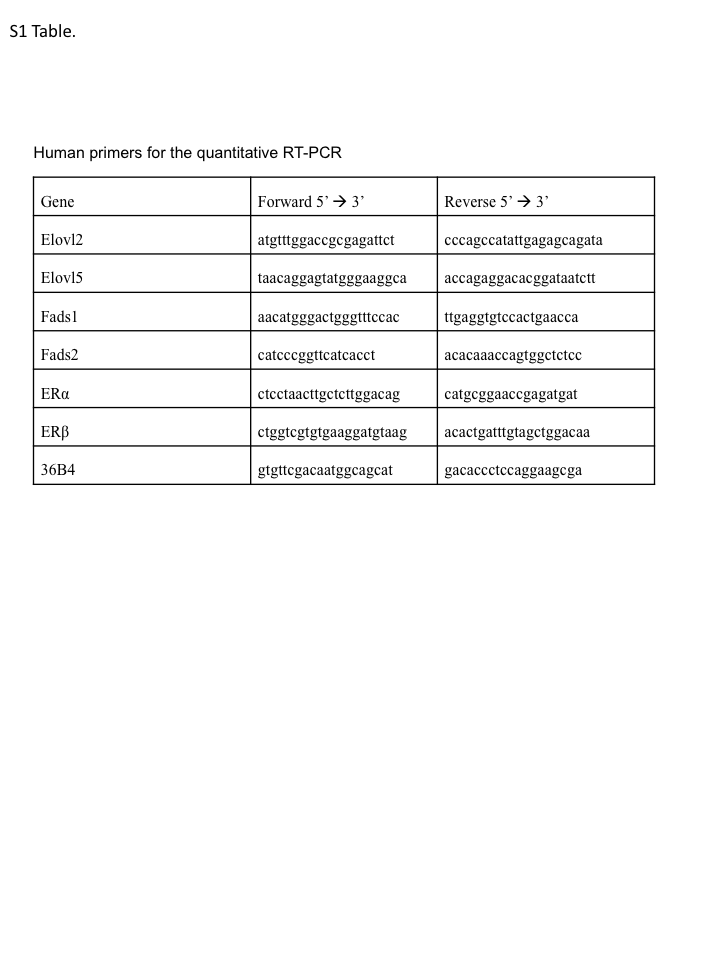

Supplement: S1 Table — (TIF) [file pone.0164241.s004.tif]
